# On the Pathology and Treatment of Spermatorhœa

**Published:** 1866-04

**Authors:** Roberts Bartholow


### ON THE PATHOLOGY AND TREATMENT OF SPERMATORRHŒA.

By ROBERTS BARTHOLOW, M.D.

Writers on spermatorrhœa have been, too often, content with repeating the observations and opinions of their predecessors. The disagreeable nature of the subject has deterred investigation, has retarded improvement in therapeutics, and has thrown the care of these cases into the hands of irregular specialists. Lallemand's treatise, which has attained an immense circulation, was written, mainly, in the interest of a special treatment. It is not a reputable book; the cases were manufactured; and the therapeutics, based upon false statements, lead to serious errors.

Spermatorrhœa is, unfortunately, very common; it produces serious mental and physical consequences, and it is most difficult to cure. It would, therefore, seem to be eminently worthy the attention and study of the philanthropic physician, the humanitarian, and, in a lower sense, of any practical doctor who would cure a disease for his own advantage.

Is spermatorrhœa a medical or surgical disease? It has usually been regarded as pertaining to the domain of surgery. This association has given rise to false views of its nature, and to improper methods of treatment. The surgeon views it as an affection of the generative apparatus, due to local causes, and to be treated by local applications. Thus, a recent surgical authority treats of it under the head of "functional disorders of the testicle." It is classified in the same category, and the treatment recommended, based upon the same principle, in other surgical works.* It is the judgment of the writer that spermatorrhœa should come under the cognizance of the physician, and that it belongs to the neuroses. This conclusion leads to practical results, as we shall see in the further consideration of this subject.

It were a labor of supererogation to enumerate the causes of spermatorrhœa. At present, we are chiefly concerned with the pathology of this affection.

The pathological conditions may be comprehended in three groups—local or genital, cerebral, spinal.

The genital consists in excessive sensibility of the sexual apparatus; the cerebral in loss or impairment of the intellectual faculties; the spinal, in loss or impairment of voluntary power, and in deranged reflex actions.

As a starting point for the inquiry, it is necessary to understand the physiology of the sexual act. The phenomena of the sexual congress belong to the class of reflex actions. The excitation of the male organ is transmitted to the cord, and the impression is reflected over the testes, vesiculæ seminales, urethra, and appended muscular apparatus, producing the seminal ejaculation. The whole constitutes the venereal orgasm. It approximates, in many of its phenomena, to epilepsy, and is accompanied in some persons with an epileptiform seizure. There is a great expenditure of nervous power in a single act of coitus or unlawful excitation, manifested by the languor, weakness, and mental feebleness which occur for a short period afterward. In how much greater degree these effects will be

* Article—Diseases of the Male Organs in Holmes' System of Surgery, p. 539, vol. iv. London: 1864. Gross' System of Surgery, pp. 831, 832, vol. ii. Philadelphia: 1865. Erichsen's Surgery, p. 1224, etc.

experienced when the orgasm is frequently repeated! That these effects are due to expenditure of nervous power, and not to the loss of seminal fluid, must be evident, when we reflect that so small a discharge from the body is, under any other circumstances, wholly incapable of producing such results. This point is confirmed by analogy; thus, a slight peripheral irritation may induce paraplegia, epilepsy, chorea, etc. It is further confirmed by the results which ensue from frequent and long-continued excitation of the genital organs, viz.:—paraplegia, epilepsy, insanity, not unfrequently, and in every case, the ready seminal flow, so-called spermatorrhœa.

I. *Genital form.*—As respects the condition of the generative apparatus, the varieties of spermatorrhœa agree. The organs are relaxed, the erections feeble, and the seminal flow watery. Irritation of the neck of the bladder is experienced; the mucous membrane of the urethra is red, injected, and exceedingly sensitive, and the testes are tender, painful, and sometimes wasted. Excitation takes place from slight causes, and the emission occurs very quickly. This circumstance is due, in a limited degree, to the hyper-sensibility of the organs themselves, but chiefly due to the state of the brain and cord—the association of ideas and the morbid excitability of the reflex functions.

There are associated with this condition of the sexual apparatus, symptoms of deranged primary and secondary assimilation, emaciation, weakness, palpitation—all of which have their origin in irregular distribution or loss of nervous force. The following is an illustrative case:—

Case I. A private patient under my charge, from long practice of masturbation, suffered with such sensitiveness of the genital organs as to have an emission upon the slightest excitement. He was pale and somewhat thin; his appetite was capricious, and he suffered much from constipation, wandering pains through the abdomen, borborygmi, and flatulence. He experienced no loss of power in his inferior extremities, neither did he suffer from any cerebral complication; he was melancholy and apprehensive of impotence, but all his mental operations were properly performed.

II. *Cerebral form.*—In the cerebral form, there is superadded to the conditions described under the genital form, certain disorders of the mind. Melancholia, delusional insanity, and dementia are the particular forms which the mental disorders assume. The statistics of any of our large insane asylums show the importance of this tendency in spermatorrhœa. Thus, in the report of Dr. O. M. Langdon, Superintendent of Longview Asylum, masturbation is assigned as the cause of insanity in forty-eight of the seven hundred and forty-seven tabulated cases.*

Case II. Was a young man admitted to St. John's Hospital as a private patient, aged about 30 years, 5 feet, 5 inches high, slightly built, and of leuco-phlegmatic temperament. His hair was scanty and beard thin. At the time of admission, he could give no connected account of himself. He was emaciated; his appetite irregular; bowels constipated. He walked unsteadily, and presented a very characteristic expression of disordered intellect. He made much of his intestinal troubles, and seemed anxious to divert my attention from the real source of his infirmities. At night, he was given up to various hallucinations; he did not sleep, but spent the night in talking in a wild and incoherent manner, and in wandering about his room making efforts to escape, to the great terror of his neighbors. From his brother, it was ascertained that he had led a wild and irregular life; that he was addicted to venereal excesses earlier in life, and lately to onanism.

Case III. Was similar to the preceding—a young man who had passed several years in the United States service, and was addicted to onanism. He was led to St. John's Hospital by a comrade, because he could not walk, and would fall down if unsupported. He presented a countenance of fatuity; he had no delirium, no hallucinations, but his "head was light," he said. He was pale, thin, and had little appetite. He admitted to me a degree of self-abuse too revolting to be narrated.

III. *Spinal form.*—The spinal form may or may not be complicated with the cerebral, but there is a distinct condition to which this term is intended to apply. In the spinal form there are present loss of power in the inferior extremities, paraplegia, incontinence of urine and fæces, and altered—diminished or perverted—and referred sensations. The following is a typical case:—

Case IV. A man, aged 35 years, came under my care, suffering from weakness, with perverted sensations of both inferior extremities. He had an ill-conditioned ulcer on the right leg; slight pressure, as of riding on horseback, induced intractable ulcerations of buttocks and thighs. He had, also, incontinence both of urine and fæces. There was some tenderness over spine in lumbar region; the tactile sensibility of the skin of legs and thighs was much diminished. He volunteered the information that earlier in life he had been much addicted to onanism, and,

*Report for 1863.

since the discontinuance of the habit, had suffered from frequent nocturnal emissions. His mental powers were unimpaired. He had the unavoidable depression and anxiety arising from his very miserable physical condition.

These typical forms may not always be clearly marked. That condition of the sexual organs described under the genital form, necessarily exists in all cases, and may or may not be accompanied by disorders of intellect and impairment of voluntary power, but is invariably accompanied by functional derangement of the cord, increased reflex excitability, and probably, also, congestion. After the excesses of venery or the practice of masturbation have ceased, this functional derangement of the cord manifests itself by repeated seminal discharges, either under the influence of slight excitement or involuntarily. It is well known that these involuntary discharges are produced in the sleeping state, under those circumstances which favor congestion of the cord, especially full suppers and lying on the back. This view is confirmed by the effects which certain lesions of the cord produce in the generative apparatus. Thus, seminal emissions frequently take place in the epileptic paroxysm, priapism in some cases, and complete abolition of the sexual appeite in other cases of disease or injury to the cord.

The relation between the sexual organs and the nervous centres is very close and important, but very obscure. The development of the organs at the age of puberty is accompanied by changes in the mental and moral nature. The sexual appetite, suddenly roused into activity at this period of life, often proves stronger than the moral control; and if the imagination is highly developed, the mind of the boy is pervaded with lascivious images—a species of delusional insanity.

A practical question here arises—What extent of involuntary seminal emission is necessary to constitute disease? Nervous persons, leading sedentary lives, are subject to tolerably frequent involuntary discharges. All healthy male adults have occasional involuntary emissions. Within certain limits these are compatible with, or, it may be, are essential to health. Persons accustomed to regular sexual intercourse are still more liable, when the habit has been interrupted, to experience these involuntary losses. When they occur not more frequently than once in two weeks, they are not indicative of disease. Patients are alarmed, also, by seeing not unfrequently after urination or defecation, a whitish tenacious discharge from the urethra, which is not seminal but mainly prostatic, and has no great significance. It occurs quite commonly after long-standing gleet, and is often misunderstood by the surgeon as well as by the patient. Frequent microscopical examinations have satisfied me as to the character of this discharge.

Another practical question in this connection is—What relation has urethritis, or the so-called irritation of the orifices of the seminal ducts, with spermatorrhœa? A local irritation of these organs transmitted to the cord may induce those lascivious dreams during which the involuntary emission usually occurs; but those irritations rarely have this effect, unless congestion of the cord or an increase of its reflex excitability have been previously induced.

If these views be correct, spermatorrhœa must take its place among the neuroses.

We may now state, in a somewhat more definite form, the conclusions to which we have arrived after a survey of the whole ground.

1. Spermatorrhæa exists in three forms or phases—genital, cerebral, and spinal.

2. The genital, the more common form, is characterized by frequent emissions with little excitement, by derangement of the primary assimilation, by greatly increased reflex excitability and, probably, congestion of the cord.

3. In the cerebral form, in addition to the preceding condition, there is present either melancholia, delusional insanity, or dementia; and

4. In the spinal form, loss of power in the inferior extremities, paraplegia, altered and referred sensations, epilepsy, etc.

The various methods of treatment which have been pursued in spermatorrhœa may be referred to either the medical or surgical order.

The medical has consisted, in the main, in efforts to improve the general health by appropriate tonics, as iron, quinine, strychnia, sea-bathing, douches; by suitable dietetics; by improved hygiene, etc.

The surgical has been directed more particularly to the state of the genital organs, which are presumed to be in a condition of disease. Hence the application of the *porte caustique* to the prostatic portion of the urethra; the use of injections of nitrate of silver, of sulphate of copper, and of acetate of lead; cold hip-bathing and injections of cold water into the rectum. In the surgical order may be classed the mechanical appliances for preventing emissions; for instance, a leather ring armed with metallic points, large enough to be worn without discomfort until an erection occurs, when the pricking will arouse the patient.

It is well known that these methods of treatment frequently fail. The medical is more successful than the surgical. Few practical surgeons, at the present day, will assent to the *dictum* of Lallemand, that "two-thirds of the cases of spermatorrhœa would be beyond the reach of medical assistance, were it not for the beneficial effects probuced by the application of the nitrate of silver to the prostatic portion of the urethra." That the *porte caustique* may be useful in a few instances, by reason of the moral effect upon the patient, is probably true. That it cures the disease by relieving the so-called "irritable state" of that part of the canal is undoubtedly incorrect. Not only does it fail to cure the patient, but the application of the caustic is often followed by most serious consequences. In all cases it produces great irritation, frequently strangury and bloody urine, and sometimes dangerous urethritis and cystitis. When the patient is relieved of these symptoms, he may confound this feeling of relief with a real improvement, to be undeceived, however, in the further progress of his case. Sometimes recovery does not take place from the effects of the application of the *porte caustique*, but a troublesome narrowing of the urethra, constituting organic stricture, remains to weary the patient and complicate the treatment.

Little can be said in favor of injections into the urethra or mechanical means to prevent the involuntary emission. They are injurious—if for no other reason—by fixing the attention of the patient upon his disagreeable infirmity; but they, also, by increasing the peripheral irritation, increase the reflex excitability of the cord. The use of stimulant diuretics, such as copaiba and cubebs, need only be mentioned to be condemned.

The medical treatment, the writer ventures to assert, frequently fails, because it does not enter sufficiently into that condition of the nervous centres, upon which the spermatorrhœa mainly depends.

In the management of this affection, it is necessary that we keep in view the pathological states pertaining to its several phases.

In the genital form of spermatorrhœa, there are associated with the peripheral irritation, increased reflex excitability and derangement of the primary assimilation. There may be more or less emaciation or plethora; more usually the former. The therapeutical methods are indicated in this brief pathological summary. The peripheral irritation and the reflex excitability must be diminished. To accomplish this important object, the continued drafts of nervous force consequent on venereal excitement must be suspended. The patient should cease his excesses whatever they may have been, and avoid all sources of excitement, whether the reading improper publications, suggestive novels or works upon his own malady, or the society of women of easy virtue. The liberties between the sexes permitted by custom in many places and in certain circles of society, without extending so far as the gratification of desire, are so very injurious to young men with "seminal weakness" that they should be absolutely interdicted. The mind of the patient, should, as far as possible, be freed from any association with his infirmity. Hence constant and agreeable employment, physical rather than mental, or an association of the two, is especially desirable. The patient may be much aided in the execution of these plans for his improvement by the administration of anaphrodisiacs, of which the bromide of potassium is, incomparably, the chief. To a man suffering under the genital form, twenty to forty grains of the bromide may be given every night until sexual desire is entirely suspended. The quantity of the remedy, which will be required to accomplish this result, will vary in different individuals. At the same time the general condition of the patient must not be neglected. If he is plethoric, his digestion active, and his bowels constipated, he will be benefited, if somewhat reduced by saline cathartics, by *liquor postassæ*, by a less liberal diet, and by diminishing the number of hours devoted to sleep. It is not frequently, however, that spermatorrhœa is associated with a condition of plethora: the opposite condition or anæmia is the rule. In this latter condition the reflex excitability of the cord is so much increased that the feeblest irritation produces a prompt emission, and the frequent discharges of nervous force affect the supply to all the organs—whence come palpitation, indigestion, constipation, irritable bladder, pain in the back, debility, and mental feebleness. In this condition the bromide of potassium is indicated to diminish the reflex excitability of the cord, and to suspend the sexual appetite, and iron, quinine, and other remedies, to improve the general health.

Whether the patient be plethoric or anæmic, remedies should be employed to relieve congestion of the cord, which occurs to a greater or less extent in every case. As the involuntary emissions are usually nocturnal, and occur chiefly when the patient is lying on his back, the cord being then most dependent, management of the position in bed is an important part of the treatment. He should lie upon his side, and if unable to continue in this position when asleep, should adopt some mechanical appliance to compel it. Tying the hand to the post of the bed, or the contrivance of Lallemand will accomplish this object. The remedial agents for affecting the same purpose, are still more important. In addition to the bromide of potassium, which possesses this property to a very considerable degree, *belladonnæ* and *ergot* may be employed. One-fourth of a grain of the extract of belladonna and two grains of Bonjean's ergotin, or five grains of fresh ergot, may be given three times a-day. In anæmic cases iron may be combined with the preceding remedies.

By these means the sexual appetite may be completely suspended, the morbid excitability of the reflex function and the congestion of the cord may be cured. These efforts must be continued until the irritability of the genital organs subsides. Having accomplished these objects an important duty yet remains—we must restore the patient to the proper performance of his sexual functions, and this must be accomplished in such a way that he will not lapse into his former infirmity. The doses of bromide must be diminished, and finally the remedy discontinued; the ergot and belladonna must be suspended, and tonics and special excitants must be employed. Extract of nux vomica or strychnia, with the saccharated carbonate of iron, is a combination very useful for this purpose. To control the action of the nux vomica and prevent too sudden stimulation of the cord, it is frequently desirable to combine belladonna with the strychnia and iron. However paradoxical such a combination may appear—seeing that strychnia and belladonna are, in some respects, physiologically antagonistic—it has been too frequently beneficial to be rejected on theoretical grounds. Cannabis Indica and allotropic phosphorus, are, also, useful in restoring the normal activity of the generative organs after this suspension of their actions by remedies.

During the whole period of time occupied by this treatment, certain accessories must not be disregarded. Simple but nutritious food must be taken; the supper should be light and eaten four or five hours previous to retiring, and stimulants should be avoided. Fluids and articles of food having a dieuretic action, should be sparingly used, since erections occur in consequence of a full bladder. Laxatives are indicated, if constipation exist, but not otherwise. A quart of cold water thrown into the rectum will often act more beneficially than laxatives.

In that phase of spermatorrhœa which has been denominated the cerebral, serious difficulties are encountered. In addition to that derangement of the sexual-spinal system, described under the genital form, there exists the complication of mental derangement. If this derangement have proceeded no further than melancholia or delusional insanity, something may be done to arrest its progress and restore the *mens sana in corpore sano.* If dementia or complete fatuity have resulted, the case may be considered nearly or quite hopeless. A smaller number of these cases would probably find their way into our asylums, if physicians generally paid more attention to the moral, mental, and remedial management of them in their incipiency. The difficulty in these cases arises from the fact that no moral influences can be used to interrupt the habit of abuse. The mechanical and other appliances which have been employed for this purpose, are merely temporary expedients which more usually fail than succeed.

It is in these psychical affections dependent upon abuse that the bromide of potassium manifests peculiar powers. Under the use of this remedy the reason frequently gains control of the disordered imagination. Beside, by destroying desire, it does away with the temptation to repetitions of abuse. When this result has been accomplished, the chain of morbid impressions must be still further interrupted by change of scene and agreeable occupation, especially physical employment. Until the treatment has proceeded so far, it will not be desirable to use those means which have been suggested for diminishing the reflex excitability and for restoring the perverted functions.

In the spinal form of spermatorrhœa, which, as exhibited in the illustrative case under that head, manifests itself by loss of power, altered and referred sensations, paraplegia, etc., there is probably structural altercation of the cord, notwithstanding, the derangement may appear to be merely reflex. So long continued functional derangement, would certainly result in organic change. In this condition of the cord, whether reflex or centric, it is not prudent to administer the bromide of potassium, which in large doses has produced, as one of its physiological actions, loss of power and paralysis of inferior extremities. Strychnia is still more objectionable if the lesion be centric. The remedies which have been most effectual in this condition are belladonna, cimicifuga, nitrate of silver, etc. The extract of belladonna may be used in a mixture with fluid extract of cimicifuga. This prescription is applicable to a less advanced stage of the disease. When paraplegia occurs the nitrate of silver is an appropriate remedy, but the tendency of this salt to be deposited in the skin, producing an unpleasant discloration, must not be forgotten. If structural change has been arrested, or the paraplegia be merely reflex, then faradisation, strychnia, and other remedies appropriate to such a state may be used with advantage.

In the typical cases presented in illustration of the several phases of spermatorrhœa, the treatment inculcated in this paper has been pursued. It may be useful to state the result. In the first case, the nightly emissions have been reduced to one in a period of about ten days; his general health is much improved, and his mind elevated from a state of despondency to cheerfulness and a confident expectation of speedy recovery. Case II. has been discharged cured, after two months' stay in the hospital. His treatment consisted in the administration every night of forty grains of the bromide of potassium, and three times a-day a pill containing one-fourth of a grain of extract of nux vomica, three drops of fluid extract of ergot or one-fourth of a grain of extract of belladonna, and three grains of saccharated carbonate of iron. His delirium and hallucinations ceased on the second night after commencing these remedies. Case III. left the hospital after ten days' stay, relieved, but not cured, and Case IV. is travelling in pursuance of his business and may now be considered well. At last report, his symptoms had disappeared, and he was restored to a state of health altogether unusual in his experience.

I have not, thus far, alluded to some of the moral and social questions which belong to this subject. The physician is not unfrequently desired to give an opinion as to the propriety of marriage of young men suffering from spermatorrhœa. A wise judgement is not easily made up on this question. Attention to the following rules, will, at least facilitate a solution of the problem:—

1. If the spermatorrhœa exist in the cerebral or spinal form, marriage is improper.
2. Marriage is proper in the genital form, if the marriage rite may be consumated, although imperfectly.
3. Marriage should never be recommended as a curative means.

There can be no doubt that judicious sexual intercourse will prove curative in the genital phase, provided that the reflex excitability of the cord be not so great as to prevent intromission of the male organ. Disappointment and unhappiness almost invariably result when marriage is resorted to as a remedy.

The management of youths, addicted to masturbation, and the treatment of impotence, pertain to this subject, but are omitted for want of space. The case of the former appeals strongly to our philanthropy and is worthy of our best efforts; the latter is not without importance in a moral and social point of view. In a future number I may discuss these subjects at length corresponding to their importance.—*Cincinnati Journal of Medicine.*

